# A Novel Method and System Implementation for Precise Estimation of Single-Axis Rotational Angles

**DOI:** 10.3390/s24175795

**Published:** 2024-09-06

**Authors:** Qinghua Yang, Yang Shen, Xuetao Sun, Changfa Wang

**Affiliations:** 1 School of Mechatronic Engineering and Automation, Shanghai University, Shanghai 200444, China; shenyang8123@163.com (Y.S.); 201339930@shu.edu.cn (X.S.); changfa1999@163.com (C.W.); 2 Shanghai Aircraft Manufacturing Co., Ltd., Shanghai 200436, China

**Keywords:** angle measurement, calibration, inertial measurement unit, axis–angle pair

## Abstract

Accurately estimating single-axis rotational angle changes is crucial in many high-tech domains. However, traditional angle measurement techniques are often constrained by sensor limitations and environmental interferences, resulting in significant deficiencies in precision and stability. Moreover, current methodologies typically rely on fixed-axis rotation models, leading to substantial discrepancies between measured and actual angles due to axis misalignment. To address these issues, this paper proposes an innovative method for single-axis rotational angle estimation. It introduces a calibration technique for installation errors between inertial measurement units and the overall measurement system, effectively translating dynamic rotational inertial outputs to system enclosure outputs. Subsequently, the method employs triaxial accelerometers combined with zero-velocity detection technology to estimate the rotation axis position. Finally, it delves into analyzing the relationship between quaternion and axis–angle, aimed at reducing noise interference for precise rotational angle estimation. Based on this proposed methodology, a Low-Cost, a High Accuracy Measurement System (HAMS) integrating sensor fusion was designed and implemented. Experimental results demonstrate static measurement errors below ±0.15° and dynamic measurement errors below ±0.5° within a ±180° range.

## 1. Introduction

In the fields of aerospace, robotics, and automotive engineering, accurately measuring the rotational angles of movable surfaces is crucial [1,2,3]. This measurement aids in understanding the system’s posture and status, enabling appropriate operations and adjustments. In aviation, precise measurement of aircraft control surface deflection is key for maintaining stability during high-speed flight, and any deviations from the designed angle measurement system can significantly impact the aircraft’s performance and safety [4,5]. In robotics, accurately measuring joint rotation is essential for executing complex movements and delicate operations [6,7]. For example, during precision assembly or surgical assistance, robots must precisely control each joint of their mechanical arm to ensure accuracy and safety [8,9]. Similarly, in automotive engineering, precise measurement of wheel steering angles and suspension adjustments is vital for maintaining vehicle stability and optimizing the driving experience. Significant measurement errors can reduce overall vehicle performance and, in severe cases [10], threaten driving safety. Hence, accurate angle measurement provides a fundamental basis for the system’s stability, accuracy, and safety.

Inertial sensor-based motion tracking methods have become a research focus in rotational angle measurement due to their low cost, compact size, and ease of integration. Inertial Measurement Unit (IMU) typically include a gyroscope, accelerometer, and magnetometer. By combining data from these sensors, attitude angles are obtained through attitude estimation algorithms [11,12,13,14,15]. IMUs reference their own coordinate system when measuring angular acceleration and velocity, making this approach to posture estimation largely unaffected by interference and obstructions [16]. In theory, they can capture the motion of a target without any limitations, leading to their widespread application [17,18,19].

In the field of rotational axis estimation, researcher Sara [20] introduced the Simultaneous Orthogonal Rotations Angle (SORA) method. This approach uses gyroscope outputs to determine the rotational angle and axis of single-axis movements. Its simplicity makes it suitable for general angular positioning, marking significant progress in angle estimation. However, it has limitations, relying solely on gyroscopes, which suffer from drift issues that worsen over time. Furthermore, the model assumes constant angular velocity, which might struggle with complex and unpredictable motion dynamics. To address gyroscopic drift, Cao [21] proposed a method for measuring spatial rotation angles using dual-axis MEMS tilt sensors. The method calculates the rotation angles of an object in space by establishing and solving model equations. A perpendicularly mounted single-axis tilt sensor is added to the original dual-axis tilt sensors, forming a three-axis tilt sensor system that effectively corrects and measures the spatial rotation angles of the object. Wang [22] proposed using an accelerometer to estimate the rotation axis can improve the precision of static measurements. However, when using Euler angles to represent rotation, the measurement precision decreases when the measurement angle exceeds 60 degrees. Subsequently, Xu [23] proposed a method for estimating the rotation axis based on incremental quaternions. This method employs incremental quaternions to calculate the rotation axis by computing intermediate rotation angles, ultimately determining the rotation angle. It effectively circumvents the gimbal lock issue associated with Euler angles and enables full-range rotation angle measurement and complete attitude estimation of the rotation axis. While it has been proven effective in handling the static motion of targets, over-reliance on quaternions has resulted in increased accumulated errors over time in computations.

The remainder of the paper is organized as follows. Section 2 lays out the coordinate systems and introduces a calibration technique designed to correct installation errors between the Inertial Measurement Unit (IMU) and the entire measurement system, effectively translating the dynamic rotation outputs of the IMU into the system frame. Section 3 presents a method for estimating the direction of the rotation axis using a tri-axial accelerometer and zero-velocity detection technology and analyzes the transformations between quaternions and axis–angle pairs. Section 4 provides experimental validation of the algorithm’s effectiveness. Finally, conclusions are summarized in Section 5.

## 2. Modeling

The coordinate systems are defined as follows. Earth coordinate system *O*, $O_{x}$ and $O_{y}$ lies in the horizontal plane, with $O_{x}$ pointing east and $O_{y}$ pointing north. $O_{z}$ is perpendicular to the horizontal ground and points upward.

The inertial measurement unit coordinate system, *I*, corresponds to the three axes of the accelerometer’s sensitive axis.

The HAMS coordinate frame, *S*, has its geometric center as the origin. The *x*-axis points forward, the *y*-axis points to the right, perpendicular to the *x*-axis, and the *z*-axis is perpendicular to the plane formed by *x* and *y*.

**Figure 1:**
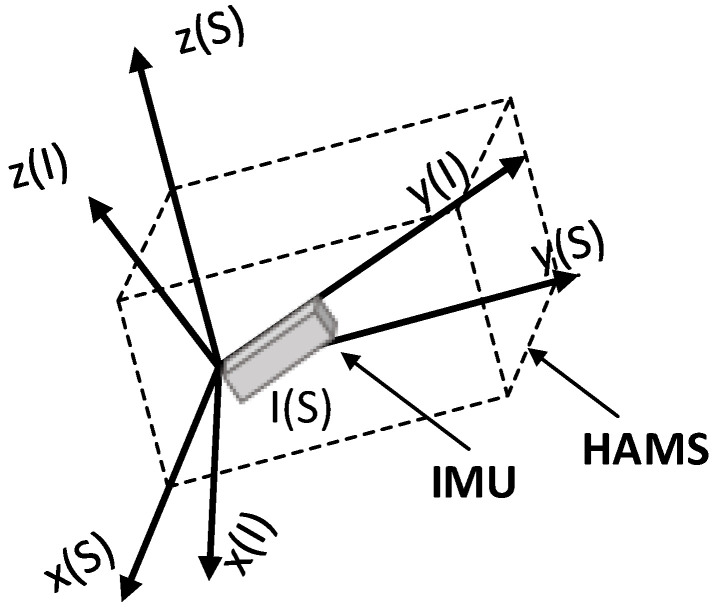
The relationship between the HAMS coordinate frame *S* and the IMU coordinate system *I*.

The relationship between the system coordinate system and the IMU coordinate system as shown in Figure 1.

The inertial measurement unit is placed inside the system, and its measurement axis has a small angular error with the outer frame of the system. Therefore, need to calibrate the small angle error between the system coordinate S and the inertial measurement unit coordinate system *I*, and convert the dynamic rotational inertial measurement output to the output of the overall. The relationship between the acceleration in the system coordinate system and the IMU coordinate system can be inferred as Formula (1).

(1) $$a^{S}=R_{I}^{S}a^{I}$$

$R_{I}^{S}$ denotes the rotation matrix of the IMU coordinate system *I* rotated to the HAMS coordinate frame *S*.

With the accelerometer stationary, rotate around the x-axis of the system. According to Formula (1), the following can be derived:

(2) $$v_{x}^{S}=R_{I}^{S}v_{x}^{I}={\left[\begin{array}{c} 1\;0\;0 \end{array}\right]}^{T}$$

where $v_{x}^{S}$ and $v_{x}^{I}$ represent the coordinates of the rotation axis *x* in the HAMS coordinate system *S* and the inertial measurement unit coordinate system *I*, respectively.

At the initial moments, we can obtain the initial matrix in the HAMS coordinate system *I*:

(3) $$R\left(\theta_{t},v_{x}^{S}\right)=\left[\begin{array}{ccc} 1 & 0 & 0\\ 0 & \cos\theta_{t} & \sin\theta_{t}\\ 0 & -\sin\theta_{t} & \cos\theta_{t} \end{array}\right]$$

Then, at time *t*, assuming that $a_{0}^{S}=\left[a_{x_{0}}^{S},a_{y_{0}}^{S},a_{z_{0}}^{S}\right]$, the acceleration in the HAMS coordinate system can be expressed as:

(4) $$\begin{array}{cc} a_{t}^{S} & =R\left(\theta_{t},v_{x}^{S}\right)a_{0}^{S}\\ \; & =\left[\begin{array}{c} a_{x_{0}}^{S}\\ a_{y_{0}}^{S}\cos\theta_{t}+a_{z_{0}}^{S}\sin\theta_{t}\\ -a_{y_{0}}^{S}\sin\theta_{t}+a_{z_{0}}^{S}\cos\theta_{t} \end{array}\right] \end{array}$$

With the angle changes, the rotation trajectory is a plane perpendicular to $v_{x}^{S}$. The $a_{t}^{S}$ perpendicular to $v_{x}^{S}$ can be obtained at any time by cross product, which is in the same direction as the rotation axis.

After three rotations $a_{t_{11}}^{S}$, $a_{t_{12}}^{S}$, $a_{t_{13}}^{S}$ can be obtained three time instants. Then,

(5) $$v_{x}^{S}=\pm \frac{\left[a_{t_{12}}^{S}-a_{t_{11}}^{S}\right]\times \left[a_{t_{13}}^{S}-a_{t_{12}}^{S}\right]}{∥\left[a_{t_{12}}^{S}-a_{t_{11}}^{S}\right]\times \left[a_{t_{13}}^{S}-a_{t_{12}}^{S}\right]∥}$$

According to Equation (2), we can obtain:

(6) $$v_{x}^{I}=\pm \frac{\left[a_{t_{12}}^{I}-a_{t_{11}}^{I}\right]\times \left[a_{t_{13}}^{I}-a_{t_{12}}^{I}\right]}{∥\left[a_{t_{12}}^{I}-a_{t_{11}}^{I}\right]\times \left[a_{t_{13}}^{I}-a_{t_{12}}^{I}\right]∥}$$

According to Equation (6), the first column vector of $R_{I}^{S}$ can be obtained, which is $v_{x}^{I}$.

Next, repeat the above steps to obtain the other two column vectors of the rotation matrix $R_{I}^{S}$, which is $v_{y}^{I}$ for rotation about the *y* axis, and $v_{z}^{I}$ for rotation about the *z* axis.

(7) $$v_{y}^{I}=\pm \frac{\left[a_{t_{22}}^{I}-a_{t_{21}}^{I}\right]\times \left[a_{t_{23}}^{I}-a_{t_{22}}^{I}\right]}{\left|\left|\left[a_{t_{22}}^{I}-a_{t_{21}}^{I}\right]\times \left[a_{t_{23}}^{I}-a_{t_{22}}^{I}\right]\right|\right|}$$

(8) $$v_{z}^{I}=\pm \frac{\left[a_{t_{32}}^{I}-a_{t_{31}}^{I}\right]\times \left[a_{t_{33}}^{I}-a_{t_{32}}^{I}\right]}{\left|\left|\left[a_{t_{32}}^{I}-a_{t_{31}}^{I}\right]\times \left[a_{t_{33}}^{I}-a_{t_{32}}^{I}\right]\right|\right|}$$

According to the definition

(9) $$v^{I}=\left[v_{x}^{I},v_{y}^{I},v_{z}^{I}\right]$$

(10) $$v^{S}=\left[v_{x}^{S},v_{y}^{S},v_{z}^{S}\right]$$

(11) $$v^{S}=R_{I}^{S}\cdot v^{I}=I_{3}$$

Therefore, according to Formulas (6)–(11) the small angle transform matrix from the inertial unit to the device frame $R_{I}^{S}$ can be obtained:

(12) $$R_{I}^{S}={\left[v_{x}^{I},v_{y}^{I},v_{z}^{I}\right]}^{-1}$$

This section has derived the coordinate transformation matrix from the IMU coordinate system to the HAMS coordinate frame, which allows for the conversion of outputs from the inertial measurement unit into the system coordinate framework.

## 3. Algorithm Design

**Figure 2:**
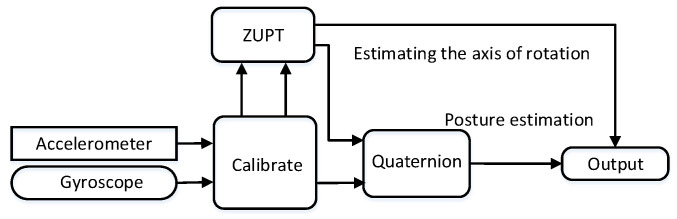
The block diagram of the algorithm system.

The entire angle measurement process involves estimating the rotation axis direction to construct the prediction model and the measurement model, and using the relationship between quaternions and axis–angle pairs to calculate the rotation angle. Figure 2 shows the block diagram of the angle measurement algorithm.

### 3.1. Zero Velocity Update

When using accelerometers for posture estimation, the precision of angle measurement by the accelerometer can be affected by other accelerations. Given the limitations of its dynamic performance, accurately identifying a static state becomes crucial [24]. Therefore, ZUPT technology is applied to determine whether an object is in a stationary state.

In the ZUPT algorithm, Wagstaff [25] proposed an adaptive threshold detector that optimizes the zero-velocity threshold by analyzing the characteristics of acceleration and angular velocity data under different motion patterns. This method can dynamically adjust the threshold according to changes in the motion state, adapting to various motion conditions and environments.

Considering that the main focus of this study is the rotation of a movable surface around a fixed axis, including both uniform and variable-speed rotation, the determination of thresholds may vary between the uniform and variable-speed rotation phases. Therefore, we propose a method of adaptive zero-velocity detection based on accelerometers and gyroscopes.

In the initial production stage, a segment of stationary time is collected for both accelerometer and gyroscope data. The magnitudes of the collected acceleration and angular velocity data, denoted as $\left|a_{k}\right|$ and $\left|\omega_{k}\right|$, respectively, are calculated. Initial thresholds thamin, thamax, thωmin, and thωmax are set. The variance of the collected acceleration magnitude, $\zeta_{a}^{2}=\mathrm{var}t_{k}\left(\left|a_{k_{t}}\right|\right)$, is computed, and an initial threshold ${\mathrm{th}}_{\zeta_{a}}$ is established.

For a stationary state, for both the accelerometer and gyroscope, their thresholds and variances satisfy:

(13) $$\;{\mathrm{th}}_{\mathrm{amin}}\leq \left|a_{k}\right|\leq {\mathrm{th}}_{\mathrm{amax}}$$

(14) $$\zeta_{a}^{2}<{\mathrm{th}}_{\zeta_{a}}$$

(15) $$\left|\omega_{k}\right|\leq {\mathrm{th}}_{\omega max}$$

Table 1 shows the initial threshold settings.

where $\zeta_{a}^{2}$ is the variance is measured for the acceleration within a time window of length seconds.

In subsequent stages, accelerometer data $a_{k}$ and gyroscope data $\omega_{k}$ are collected in a loop, where *k* represents the time step index. The most recent N samples constitute a sliding window ak−N+1,…,ak and ωk−N+1,…,ωk. For each sample, the acceleration error term is calculated as $e_{k}={\left|ak-g\right|}^{2}$, and the gyroscope error term as $f_{k}={\left|\omega k\right|}^{2}$. The weighted mean of errors within the sliding window is computed as $S_{k}=\frac{1}{N}\sum {i=k-N+1}^{k}\left(\frac{1}{\sigma_{a}^{2}}e_{i}+\frac{1}{\sigma \omega^{2}}f_{i}\right)$. For data samples satisfying the stationary status, the acceleration error term $e_{k}$ and gyroscope error term $f_{k}$ are calculated. Using maximum likelihood estimation, the distribution parameters of the acceleration and gyroscope error terms, namely $\mu_{e}$, $\sigma_{e}^{2}$, $\mu_{f}$, and $\sigma_{f}^{2}$, are estimated. Based on the estimated parameters, thresholds ${\mathrm{th}}_{\mathrm{amin}}$, ${\mathrm{th}}_{\mathrm{amax}}$, and ${\mathrm{th}}_{\omega \max}$ are updated to adapt to the current motion state.

**Figure 3:**
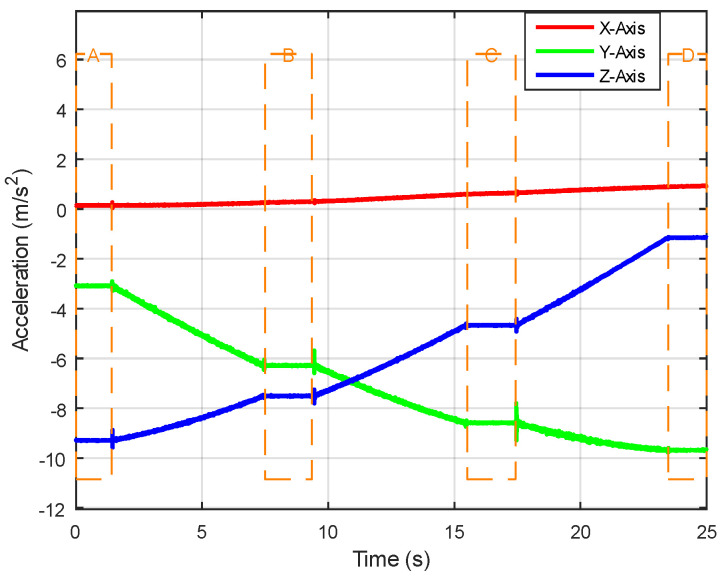
Changes in acceleration data during stillness and motion.

**Figure 4:**
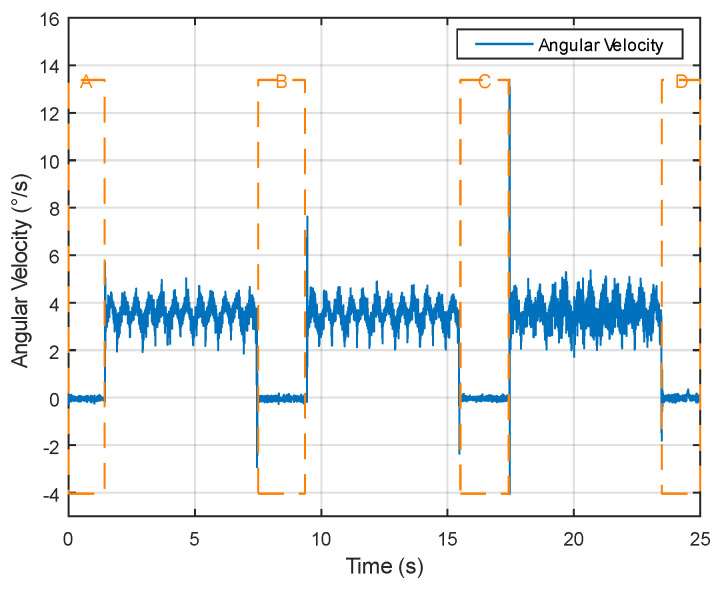
Changes in angular velocity data during stillness and motion.

**Figure 5:**
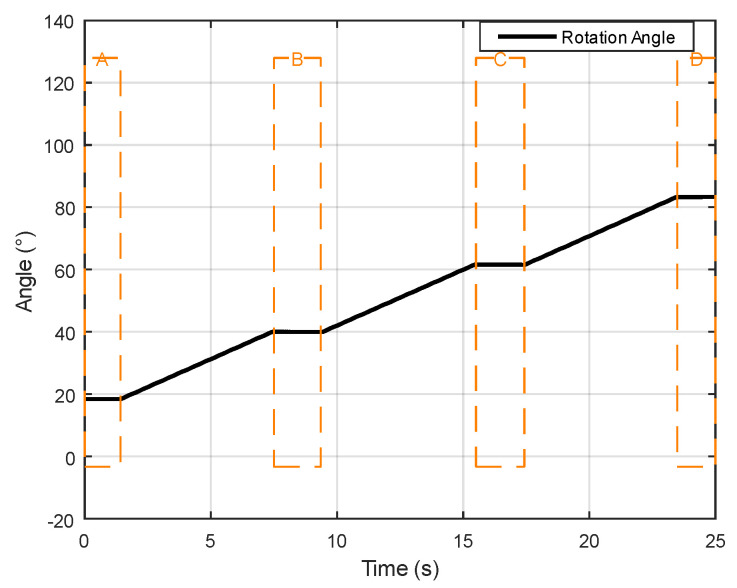
Changes in angular data during stillness and motion.

Selecting a sample that transitions from a stationary state to uniform rotation and then back to a stationary state, Figure 3 illustrates the variation in acceleration data, with fluctuations observed over a specific time period. Areas of low fluctuation indicate the object’s stationary state.The part enclosed by the dashed box in the figure. Displaying the corresponding changes, Figure 4 reveals areas of low fluctuation in gyroscope data, corresponding to the presence of a stationary state, similar to the acceleration data. Directly presenting the rotation angles calculated after data processing, Figure 5 clearly shows that the angle changes corresponding to the stationary state are very small, intuitively demonstrating the working principle and effect of ZUPT.

### 3.2. Estimating the Rotation Axis

In the study of angle estimation for single-axis rotational motion, accurate estimation of the rotational axis’s attitude is a fundamental and critical step. Currently, most data fusion algorithms are based on a fixed and visible rotation axis, imposing stringent requirements on the installation of angle measurement devices. Therefore, this section proposes a method for estimating the rotation axis that does not depend on the installation position, effectively avoiding the impact of installation errors.

**Figure 6:**
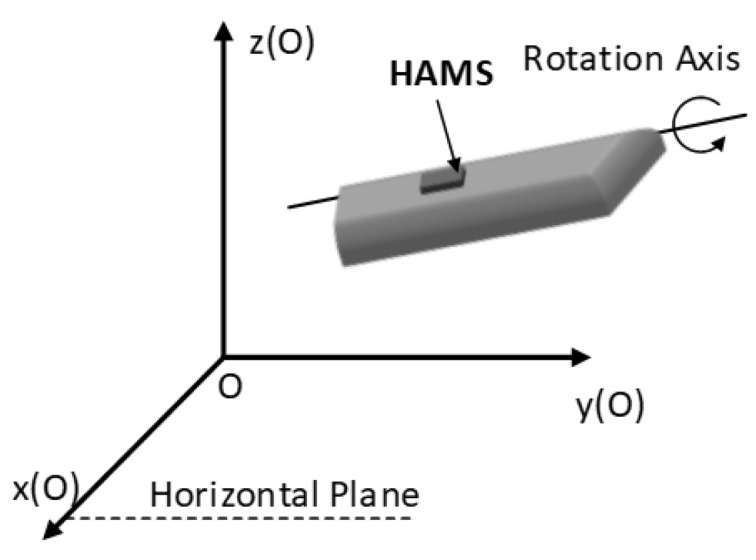
Schematic of the rotation axis direction in the geodetic coordinate system.

Define the z-axis of the moving plane coordinate system *C* as the axis of rotation, as shown in Figure 6.

$v^{C}$ and $v^{S}$ represent the directions of the rotation axis in the moving plane coordinate system *C* and in the HAMS coordinate system *S*, respectively.

(16) $$\begin{array}{c} v^{C}=R_{S}^{C}v^{S}={\left[\begin{array}{ccc} 0 & 0 & 1 \end{array}\right]}^{T} \end{array}$$

When the accelerometer is in a stationary state, the initial matrix of the moment of the moving plane rotating around the rotation axis can be obtained at the initial moment:

(17) $$R\left(\theta_{t},v^{C}\right)=\left[\begin{array}{ccc} \cos\theta_{t} & -\sin\theta_{t} & 0\\ \sin\theta_{t} & \cos\theta_{t} & 0\\ 0 & 0 & 1 \end{array}\right]$$

Then, after a time *t*, the acceleration can be expressed as:

(18) $$\begin{array}{cc} a_{t}^{C} & =R\left(\theta_{t},v^{C}\right)a_{0}^{C}\\ \; & =\left[\begin{array}{c} a_{x_{0}}^{C}\cos\theta_{t}-a_{y_{0}}^{C}\sin\theta_{t}\\ a_{x_{0}}^{C}\sin\theta_{t}+a_{y_{0}}^{C}\cos\theta_{t}\\ a_{z_{0}}^{C} \end{array}\right] \end{array}$$

Similarly to the aforementioned calibration method, based on Formulas (2)–(5), rotating the moving plane around the rotation axis three times can yield $v^{S}$, which is the direction of the rotation axis in the HAMS coordinate system.

(19) $$v^{S}=\pm \frac{\left[a_{t_{2}}^{S}-a_{t_{1}}^{S}\right]\times \left[a_{t_{3}}^{S}-a_{t_{2}}^{S}\right]}{|\left[a_{t_{2}}^{S}-a_{t_{1}}^{S}\right]\times \left[a_{t_{3}}^{S}-a_{t_{2}}^{S}\right]|}$$

### 3.3. Attitude Solution

In the field of attitude estimation, Hamilton quaternions are widely used to represent rotational states. Theoretically, axis–angle pairs can be derived from quaternions, leading to the calculation of rotation angles. However, rotation angles derived directly from a single element of a quaternion often fail to meet the accuracy required for practical applications, because using a single element from the quaternion to solve for the rotation angle does not accurately reflect the relationship between the rotation angle and the quaternion, and the estimation of the rotation angle is highly sensitive to the rotation axis [26]. Therefore, this section will delve into the analysis of the relationship between quaternions and axis–angle pairs, aiming to achieve precise estimation of rotation angles by reducing noise interference.

This paper utilize Hamilton quaternions q to represent the state of rotation. A quaternion is composed of a real number and a vector, represented as follows:

(20) $$\begin{array}{cc} q & ={\left[\begin{array}{cccc} q_{0} & q_{1} & q_{2} & q_{3} \end{array}\right]}^{T}\\ \; & ={\left[\begin{array}{cccc} \cos\frac{\theta }{2} & v_{x}\sin\frac{\theta }{2} & v_{y}\sin\frac{\theta }{2} & v_{z}\sin\frac{\theta }{2} \end{array}\right]}^{T} \end{array}$$

In this representation, $q_{0}$ denotes the real number, while $u={\left[q_{1},q_{2},q_{3}\right]}^{T}$ represents the vector component.

#### 3.3.1. Rotational Axis Transformation

Based on the rotational axis obtained from Formula (19), it needs to be converted to the rotational axis in the Earth-fixed coordinate system. Therefore, we need to utilize the initial quaternions q obtained from the system device and calculate the rotational axis in the Earth-fixed coordinate system using Formulas (21) and (22).

(21) $$\begin{array}{c} R_{O}^{S}=\left[\begin{array}{ccc} 1-2q_{2}^{2}-2q_{3}^{2} & 2q_{1}q_{2}-2q_{0}q_{3} & 2q_{1}q_{3}+2q_{0}q_{2}\\ 2q_{1}q_{2}+2q_{0}q_{3} & 1-2q_{1}^{2}-2q_{3}^{2} & 2q_{2}q_{3}-2q_{0}q_{1}\\ 2q_{1}q_{3}-2q_{0}q_{2} & 2q_{2}q_{3}+2q_{0}q_{1} & 1-2q_{1}^{2}-2q_{2}^{2} \end{array}\right] \end{array}$$

(22) $$\begin{array}{c} v^{O}=v^{S}\cdot {R_{O}^{S}}^{-1} \end{array}$$

#### 3.3.2. The Relationship between Quaternions and Axis–Angle Pairs

After filtering the obtained quaternions, calculate the angles. Substitute the rotational axis $v^{O}$ obtained from Formula (22) into Formula (20) and simplify the expression. This process yields the expressions for four rotation angles as demonstrated in Formula (23).

(23) $$\theta =2\arctan2\left(\frac{1}{3}\left(\frac{q_{\;1}}{v_{x}}+\frac{q_{2}}{v_{y}}+\frac{q_{3}}{v_{z}}\right),q_{0}\right)$$

where $v_{x}$, $v_{y}$, and $v_{z}$ are the three components of the axis of rotation pose estimation $v^{S}$.

When using the aforementioned formula, it is necessary to use the derivatives of the rotational components, and if the component of the axis of rotation is small or even 0, this will cause the estimated value of the angle to be distorted, so it is necessary to filter out the component when solving. Introduce the gain component γ.

(24) $$\begin{array}{cc} \; & \gamma_{x}=\left\{\begin{array}{c} 0,\;\mathrm{if}\;v_{x}<\xi_{v}\\ 1,\;\mathrm{otherwise}\; \end{array}\right.\\ \; & \gamma_{y}=\left\{\begin{array}{c} 0,\;\mathrm{if}\;v_{y}<\xi_{v}\\ 1,\;\mathrm{otherwise}\; \end{array}\right.\\ \; & \gamma_{z}=\left\{\begin{array}{c} 0,\;\mathrm{if}\;v_{z}<\xi_{v}\\ 1,\;\mathrm{otherwise}\; \end{array}\right. \end{array}$$

Convert Formula (23) to:

(25) $$\theta =2\arctan2\left(\frac{\gamma_{x}\frac{q_{1}}{v_{x}}+\gamma_{y}\frac{q_{2}}{v_{y}}+\gamma_{z}\frac{q_{3}}{v_{z}}}{\gamma_{x}+\gamma_{y}+\gamma_{z}},q_{0}\right)$$

where arctant2 is a function used to calculate the angle θ for any point (x,y), returning an angle value ranging from $-180^{{}^{\circ}}$ to $180^{{}^{\circ}}$.

**Figure 7:**
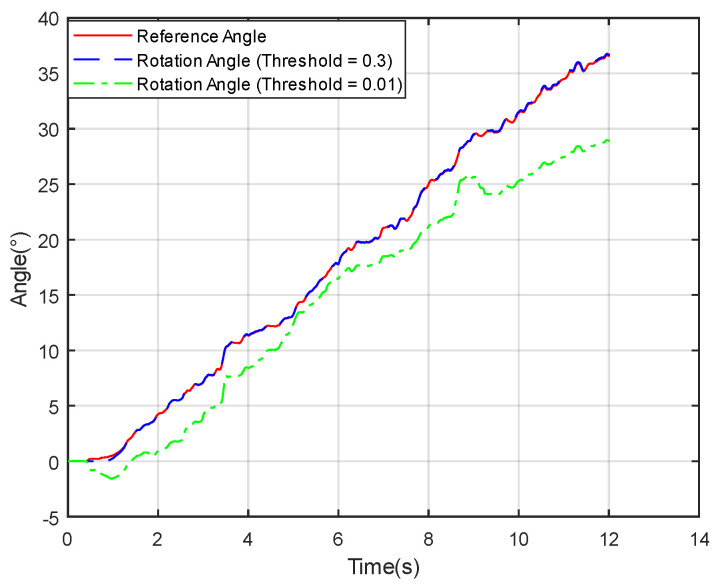
Comparison experiment of different thresholds for rotational components.

According to Formula (24), if a component of the estimated rotational axis $v^{O}$ does not meet the criterion during the process of solving for the rotation angle, that component will not be incorporated into the equation for solving the rotation angle. Appropriate thresholds need to be selected, as improper threshold selection, as shown in Figure 7, can result in significant distortions in certain angle calculations.

Formula (25) will resort to utilizing the more reliable rotational axis estimation $v^{O}$ component to solve for the rotation angle, where the calculated rotation angle becomes the final rotation angle.

## 4. Experiments and Discussions

**Figure 8:**
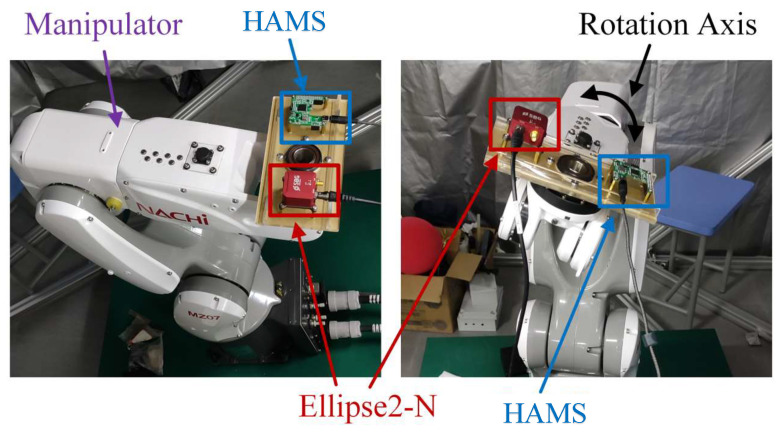
HAMS and AHRS Experimental.

In this section, the precision three-axis turntable (SGT320E from China Aviation Industry Corporation, Beijing, China) is used to simulate single-axis rotational motion. With an accuracy of 5 arcseconds, this precision three-axis turntable is highly suitable for applications requiring high-precision inertial attitude sensors, providing actual reference data for rotation angles. In both dynamic and static performance experiments, and taking the angles measured by SGT320E as references, the rotation angles calculated by the method proposed in this paper are compared with the rotation angles obtained using the IOE method [23]. To better validate the effectiveness of the proposed method in estimating rotation angles and axis attitudes during single-axis rotational motion, a low-cost high-precision angle measurement system (High Precision Angular Measurement System, HAMS) based on sensor fusion is designed and implemented. It is compared with the currently available Ellipse2-N (a compact, high-precision Attitude and Heading Reference System (AHRS)) (VectorNav: Dallas, TX, USA) in Figure 8. The Ellipse2-N device can provide measurement accuracies of 0.1° for roll and pitch, and 0.8° for yaw [27,28]. Through comparison, we can evaluate the accuracy and reliability of the proposed method.

### 4.1. Static Performance Experiment

In the research presented in this paper, static performance experiments are a crucial component, used to validate and assess the stability and accuracy of algorithms in the absence of external dynamic inputs [25]. Such experiments are particularly important for applications requiring precise angle measurements over extended periods.

**Figure 9:**
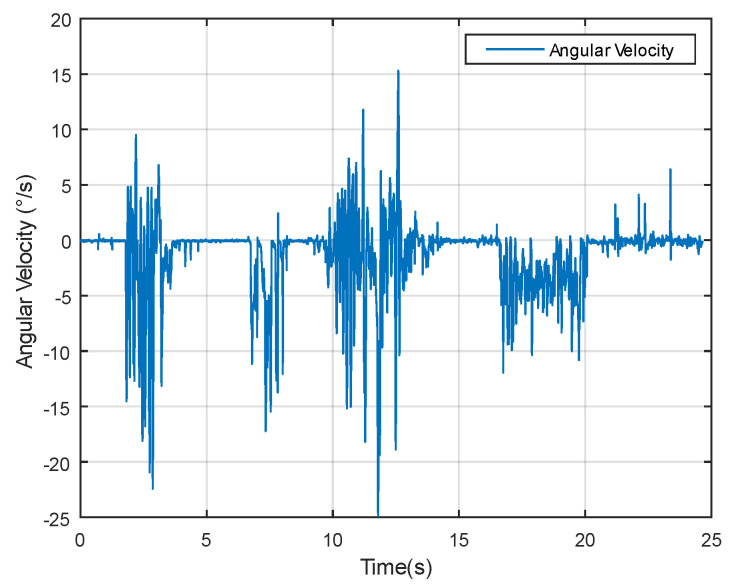
Angular velocity data in static experiment.

In static experiments, controlled disturbances are intentionally introduced during rotation to simulate uncertain factors present in reality. These disturbances are designed to take various forms, including mechanical vibrations, temperature fluctuations, and electromagnetic interference. In this study, mechanical vibration is utilized to assess the applicability and robustness of the proposed method. In each experiment, as depicted in Figure 9, disturbances are introduced after the rotation reaches the set angular velocity and gradually reduced over time until the system comes to a complete stop, allowing it to return to its static state.

**Figure 10:**
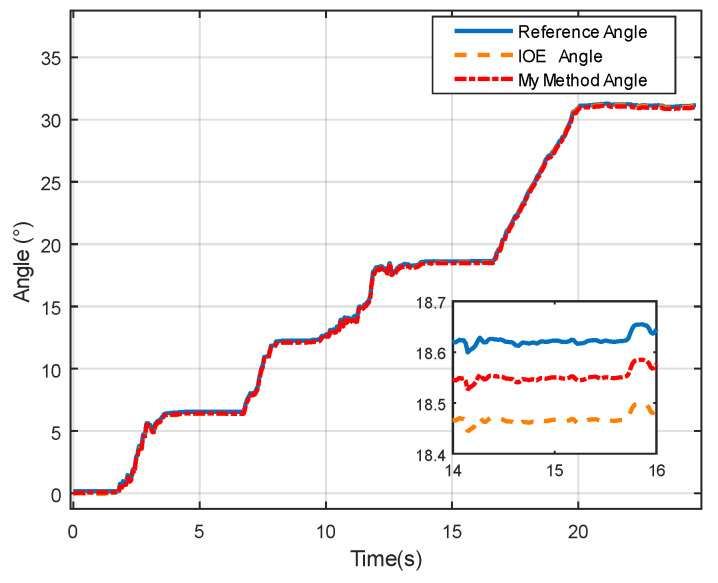
Angle comparison in static characteristic experiment.

**Figure 11:**
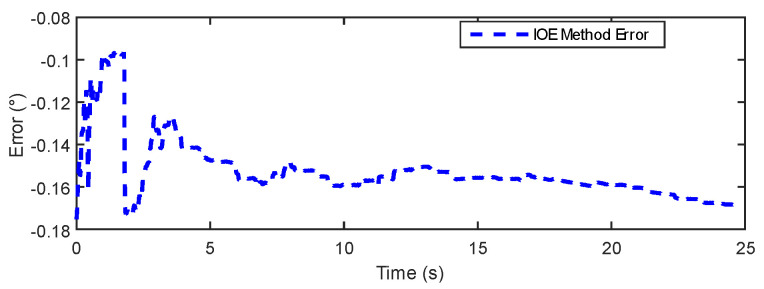
IOE error in static experiment.

**Figure 12:**
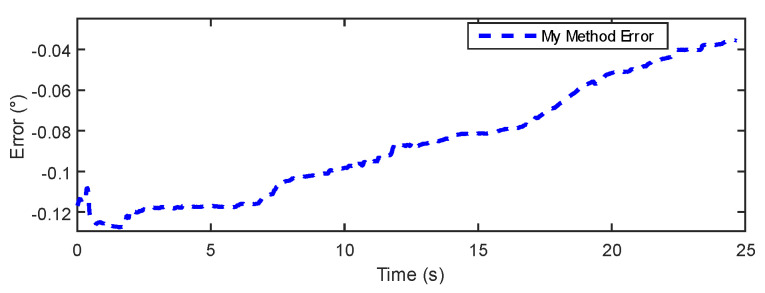
Our method’s error in static experiment.

The data comparison plot in Figure 10 demonstrates a high consistency between the proposed method and the IOE method [23] regarding angle measurements during rotation and disturbance. After the dynamic disturbance, both methods show a return to the static state, indicating that the proposed method can provide more precise measurement results. Figure 11 and Figure 12 further illustrate the measurement errors of the proposed method and the IOE method [23]. From Figure 12, it can be observed that although the proposed method initially exhibits an error greater than 0.1° at the start of rotation, the error gradually decreases over time and stabilizes, indicating good accuracy in adapting to disturbances and returning to the static baseline. In contrast, the error of the IOE method, as shown in Figure 11, quickly peaks at the beginning of the experiment, suggesting sensitivity to initial conditions. When disturbances are introduced, the system requires time to stabilize, with the final error stabilizing at around 0.16°. Table 2 provides a comparison of the static experimental data between the IOE method and the proposed method. The results show that the proposed method yields a lower error and RMSE in comparison to the IOE method.

### 4.2. Dynamic Characteristic Experiment

**Figure 13:**
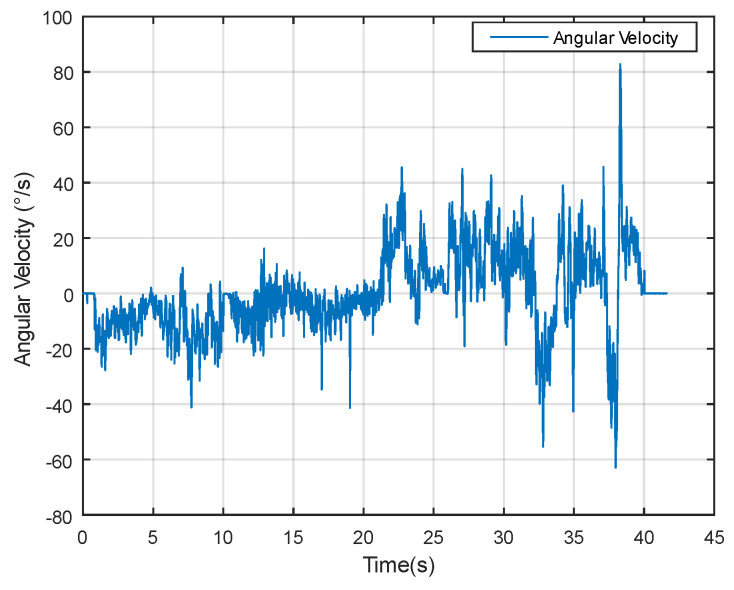
Angular velocity data in dynamic experiment.

Dynamic performance experiments involve assessing the algorithm’s responsiveness under changing temporal conditions. As evident from the angular velocity data in Figure 13, significant fluctuations occur, providing a dynamic backdrop for evaluating the measurement methods.

**Figure 14:**
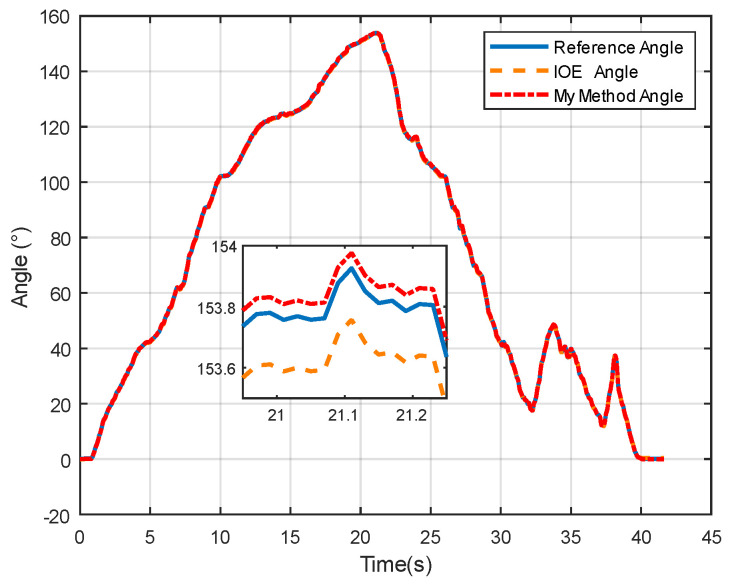
Angle comparison in dynamic characteristic experiment.

From Figure 14, it can be observed that both the proposed method and the IOE method [23] can track the true values with good consistency, indicating that the proposed method effectively captures the trend of angle changes. From the enlarged section, it can be observed when the angle reaches its peak, both the IOE method and the proposed method exhibit smaller fluctuations in estimated angles, resulting in smoother variations.

**Figure 15:**
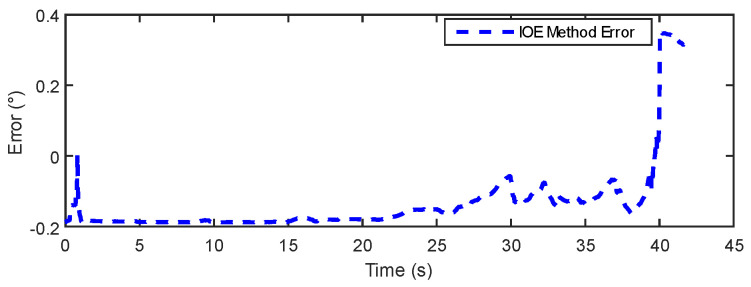
IOE error in dynamic experiment.

**Figure 16:**
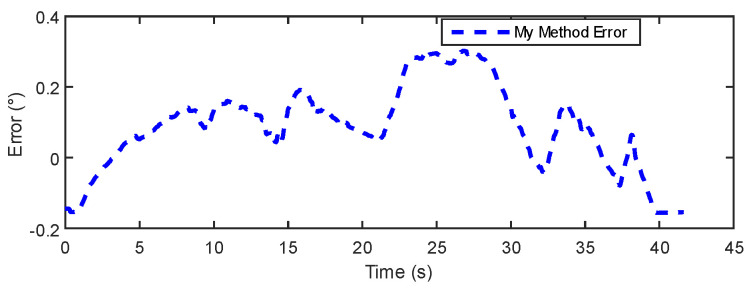
My Method error in dynamic experiment.

In Figure 15 and Figure 16, the results show that the estimation error of the IOE method remains around 0.2° for the majority of the regions, indicating good stability. However, towards the end, the IOE method exhibits a significant error spike, suggesting over-reliance on gyroscopic data and the phenomenon of gyroscopic error accumulation. The maximum estimation error reaches 0.4°. The errors of the proposed method primarily concentrate within a range of 0.3°. Table 3 provides a comparison of the dynamic experimental data between the IOE method and the proposed method. The results indicate that the proposed method exhibits some improvement in error and RMSE compared to the IOE method.

### 4.3. Experimental Features of the System

**Figure 17:**
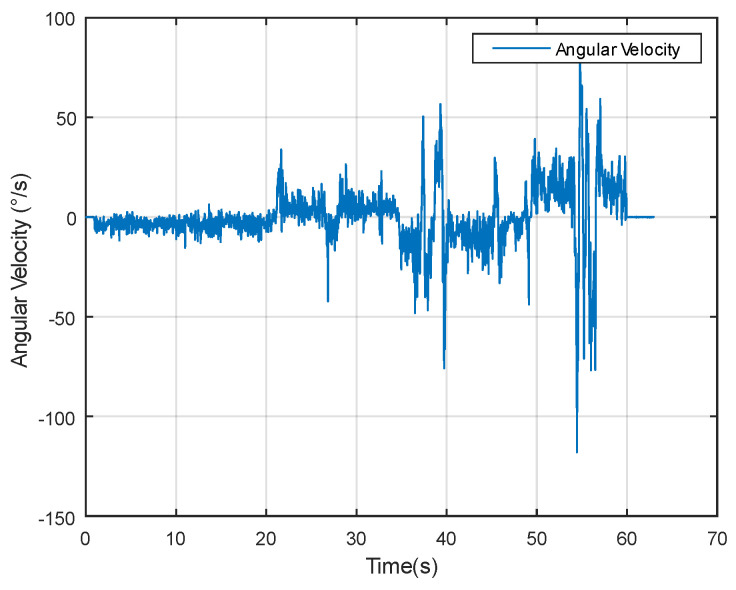
Angular velocity data in system experiment.

In the system experiment, a high-precision angular measurement system (HAMS) based on sensor fusion at low cost was designed and implemented. Angle estimation for single–axis rotation was conducted based on the method proposed in this paper, and compared with a compact, high-precision Attitude and Heading Reference System (AHRS).The angular variation data in the experiment is shown in Figure 17.

**Figure 18:**
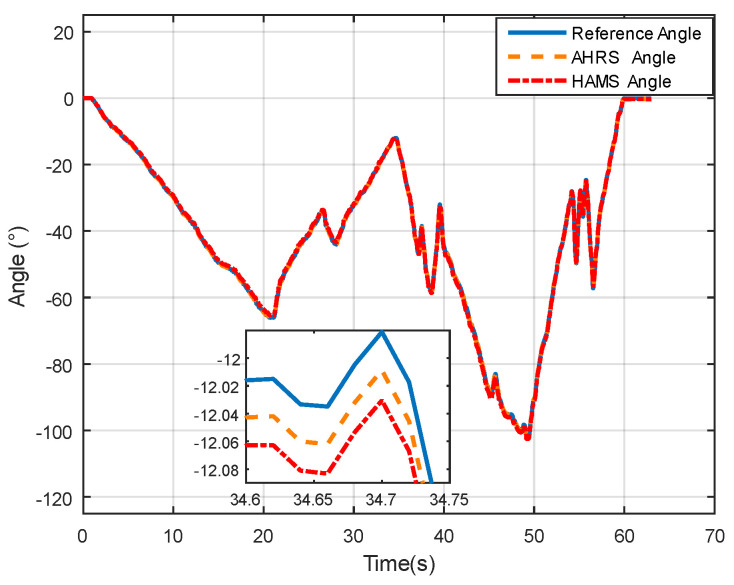
Angle comparison in system experiment.

**Figure 19:**
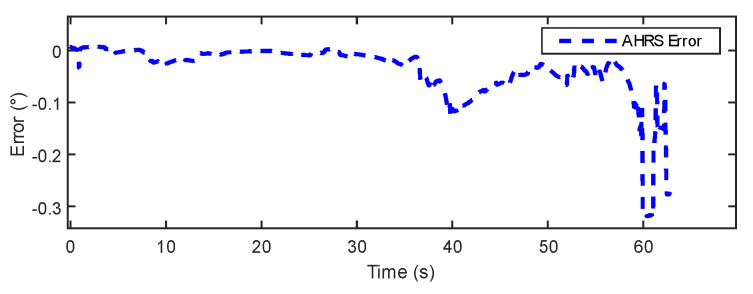
AHRS error in system experiment.

**Figure 20:**
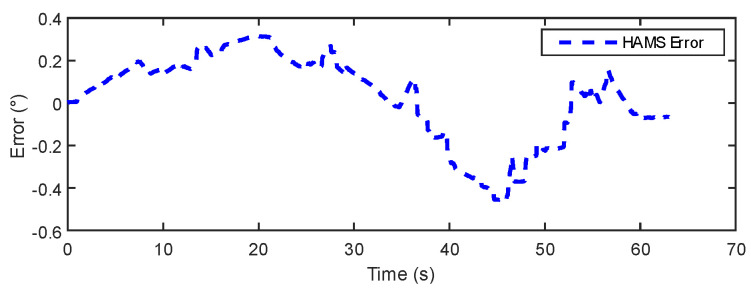
HAMS error in system experiment.

In Figure 18, the AHRS racks the reference angle more closely than the HAMS. In Figure 19 and Figure 20, the HAMS designed in this paper exhibits larger variations in error, staying within 0.5°. However, it demonstrates better performance in the final static state with smaller errors compared to AHRS, indicating the superior static performance of HAMS. The AHRS maintains relatively consistent errors of less than 0.1° for the majority of the time, but towards the end, due to the phenomenon of gyroscopic error accumulation, the error exceeds 0.3°. Table 4 lists the error and root mean square error (RMSE) data for both AHRS and HAMS. Although HAMS has higher error values, the results suggest that in certain specific scenarios, considering its low-cost design, it may still exhibit acceptable performance.

Experimental tests compared with the IOE method demonstrate the effectiveness of the approach proposed in this paper, particularly in enhancing static measurement performance. Compared to costly AHRS devices, the HAMS designed according to the method in this paper significantly reduces costs while maintaining a measurement accuracy of 0.5° for dynamic error and 0.15° for static error. This makes precise angle measurement feasible, even with limited budgets. Furthermore, the low–cost HAMS equipment demonstrates outstanding static measurement performance, which is crucial for industrial applications with stringent requirements for both cost and performance.

## 5. Conclusions

This study focuses on a novel method for estimating single–axis rotation angles for movable planes rotating around a fixed axis. Traditional angle measurement techniques are often limited by sensor constraints and environmental interference, leading to significant deficiencies in accuracy and stability. Moreover, existing methodologies typically rely on the strict alignment of the IMU’s X axis or Y axis with the rotation axis of the measured plane. However, due to installation errors, there is often a discrepancy between the assumed and actual rotation axes, resulting in a significant deviation between the measured and true angles [29].

To address the aforementioned issues, this paper proposes an innovative method for single–axis rotation angle estimation. The core innovations of this method lie in three aspects: Firstly, we introduce a novel calibration method for the installation error of the inertial measurement unit, allowing effective conversion of dynamic rotational inertial measurement data to system shell output, thereby significantly enhancing the accuracy and reliability of the data. Secondly, by utilizing a combination of triaxial accelerometers and zero velocity detection technology, we accurately estimate the direction of the rotation axis, effectively eliminating the influence of the installation position on the measurement results. This ability to negate the impact of installation position gives the method high versatility, making it adaptable to a wider range of single-axis rotational angle estimation tasks. Finally, quaternion representation of rotation combined with the estimated rotation axis is employed for attitude estimation.

In the experimental section, we first compared the rotation angles calculated using the method proposed in this paper with those obtained using the IOE method [23]. Static experiments demonstrates good system stability, with a maximum error of less than 0.15°. Dynamic experiments showed a maximum error of less than 0.3°, indicating that the proposed method effectively captures the trend of angle changes. To further validate the effectiveness of the method proposed in this paper in estimating the rotation angles and axis orientations of single–axis rotational motion, we designed and implemented HAMS, which was compared with the commercially available AHRS (Ellipse-2). The HAMS designed according to the method proposed in this paper maintains a measurement accuracy of 0.5° for dynamic errors and 0.15° for static errors, while significantly reducing costs. This makes it possible to perform accurate angle measurements within a limited budget.

## Figures and Tables

**Table 1:** Static experimental data.

Parameter Value	Value
$t_{k}$	0.005 s
${\mathrm{th}}_{\mathrm{amin}}$	9.74 m/s^2^
${\mathrm{th}}_{\mathrm{amax}}$	9.83 m/s^2^
${\mathrm{th}}_{\zeta_{a}}$	0.001 m/s^2^
${\mathrm{th}}_{\omega max}$	0.12°/s

**Table 2:** Static experimental data.

	IOE	My Method
Error (°)	0.18	0.13
RMSE (°)	0.15325	0.09065

**Table 3:** Dynamic experimental data.

	IOE	My Method
Error (°)	0.38	0.30
RMSE (°)	0.17535	0.14972

**Table 4:** System experimental data.

	AHRS	HAMS
Error (°)	0.3	0.5
RMSE (°)	0.06798	0.20662

## Data Availability

No new data were created or analyzed in this study.
